# Cochlear Implants: What the Neurosurgeon Needs to Know

**DOI:** 10.7759/cureus.29998

**Published:** 2022-10-06

**Authors:** Aisha S Obeidallah, Mousa K Hamad, Ryan M Holland, Alan R Cohen, Andrew J Kobets

**Affiliations:** 1 Neurological Surgery, Montefiore Medical Center, Moses Campus, New York City, USA; 2 Neurological Surgery, Johns Hopkins University, Baltimore, USA

**Keywords:** neuro-otology, otology, malfunction, mri, monopolar, deep brain stimulation, ventriculoperitoneal shunt, intraoperative monitoring, neurosurgery, cochlear implant

## Abstract

Patients with cochlear implants (CIs) commonly undergo neurosurgical interventions for concurrent pathologies. The neurosurgeon must be aware of the limitations these devices place on treating these patients and all pertinent interactions CIs have with common neurosurgical instruments and procedures. A literature search was performed utilizing the terms “cochlear implant” and “neurosurgery” or “neurosurgical” and all associated iterations. We reviewed the abstracts of 146 generated reports and eight published papers discussing the interaction and limitations of CI use in different neurosurgical procedures.

Five realms were identified in which a CI may potentially interfere with standard neurosurgical care: Magnetic resonance imaging (MRI), radiotherapy, deep brain stimulation (DBS), intraventricular shunt placement, and intraoperative neuromonitoring (IONM). First, MRI use with CIs is limited due to thermal injury risk, imaging disruption, and implant damage. Secondly, high-dose >50 Gy single-fraction linear accelerator-based radiosurgery has been demonstrated to result in a loss of radio frequency link range in CIs, interfering with their function. Next, during surgery for DBS, the need for MRI and microelectrode recording requires CI magnet removal by neurotology and the surgeon must communicate with a non-hearing patient. Tunneling of shunts must accommodate CI position retroauricularly, if ipsilateral, and programmable valves must be placed >2 cm from the CI to prevent interference. Intraoperative neuromonitoring may produce voltages that interfere with CIs, and while monopolar cautery may pose the same risk, no study has proven this to date. Generally, bipolar cautery is safe and favored >1 cm from CIs. MRI use is limited in CI patients, although MRI-safer devices are in production. DBS electrodes may be successfully placed after CI magnet removal. Programmable shunt valves may be placed >2 cm away from CIs and radiosurgery <50 Gy has not demonstrated harm to these devices. IONM and monopolar cautery have not been demonstrated to directly affect CIs; however, more research is needed.

## Introduction and background

Cochlear implants (CIs) were first invented in 1957 by André Djourno and Charles Eyriès. They are neuroprosthetic devices that comprise an external microphone that processes and transmits sound via electromagnetic waves transmitted to an internal component, consisting of a receiver and stimulator ending in a silicone electrode array surgically implanted into the cochlea. The function of CIs is to bypass the normal peripheral auditory system and stimulate the cochlear nerve directly. According to the National Institutes of Health (NIH), a total of 736,900 cochlear implants have been implanted worldwide as of September 2019, with 118,100 adult and 65,000 pediatric implants in the United States (US) [1]. While most CIs are done microscopically, recent advances in endoscopic technology and technique have opened opportunities for endoscopic implantation. This technique allows for visualization of the round window and safe implantation, especially in patients with difficult inner ear anatomy or severe otosclerosis while avoiding a posterior tympanostomy and decreasing the risk of seventh nerve injury [2].

The number of CIs have risen since 2012, and CI patients are more commonly undergoing neurosurgical interventions for concurrent pathologies. The neurosurgeon must be aware of the limitations these devices place on treating these patients. Our objective is to review all pertinent interactions CIs have with common neurosurgical diagnostic tests, operative instruments, and therapeutic implants.

Methodology

A literature search using PubMed was performed utilizing the terms “cochlear implant” and “neurosurgery” or “neurosurgical” and all associated iterations. The abstracts of the generated 146 reports were reviewed and eight papers specifically discussing the interaction and limitations of CI use in different neurosurgical procedures and with different neurosurgical instruments were reviewed.

## Review

Results

MRI

MRI use with CIs is limited due to thermal injury risk to the patient, imaging disruption, and potential implant damage. The external component, which is removable, generates electromagnetic waves that are transmitted to the internal component. It is usually held in place by the implanted receiver (behind the ear) via a magnet that is located below the skin. This implanted magnet creates an MRI environmental risk, is associated with a possibility of displacement, and may demagnetize or reverse the polarity of the magnetic field creating large artifacts, which will limit the utility of the study [3]. Up until recently, CIs were considered an absolute contraindication to MRI, but now many models have 1.5T and 3T conditional labeling [4]. It is recommended that a tight headwrap is applied before MRI in specification-conforming devices [5]. In a retrospective analysis of 1706 CI recipients, 21 (1.23%) patients required revision surgery due to magnet dislocation, and 10 of those cases (47.62% of the revision cases) were after 1.5T MRI with a tight compression bandage headwrap, in compliance with the manufacturer recommendations [6]. Another retrospective review of 30 MRI scans on a 1.5T scanner demonstrated that of the 18 patients, five of them exhibited extreme pain during the study, one patient had a displaced magnet after the scan, one patient required removal and reinsertion, and the last a polarity reversal [7]. The study concluded that sedation and careful head positioning may be necessary to decrease the chance of adverse events. Zhen et al. reported on a three-year-old who developed unilateral sensorineural hearing loss, had a 3T compatible CI, and required multiple MRIs for surveillance of a concurrent optic pathway glioma, all done under general anesthesia [8]. Mild tachycardia was the only notable effect during the first MRI, which was not reproduced in the subsequent three. More work needs to be performed to evaluate the safety of these devices, and neurosurgeons must always be aware of their associated risks. 

Deep Brain Stimulation (DBS)

It is unknown whether CIs may interfere with DBS implantation and performance. Buell et al. described a 70-year-old male with Parkinson’s disease and bilateral cochlear implants whose implants’ magnets were removed in order to obtain a pre-operative MRI and then replaced the same day [9]. Despite magnet removal, there was still a significant artifact making the subthalamic nucleus (STN) a more favorable target than the globus pallidus internus. The patient underwent stereotactic microelectrode recording-guided insertion of DBS leads into the STN bilaterally. During the surgery, the patient’s right cochlear implant was turned off, but the left one remained active in order to communicate during the surgery. However, both implants needed to be turned off in order to get accurate microelectrode recordings during the STN lead placement. After placement, the left implant was turned back on in order to listen to the surgeon’s instructions during testing. Following the surgery, both cochlear implants were functioning normally and the patient reported improvement in his Parkinson’s symptoms [8]. Regarding surgery for DBS, the need for MRI and microelectrode recording requires CI magnet removal by neurotology. Tunneling the distal wire must avoid the remaining CI components, and the surgeon must prepare to communicate with a non-hearing patient perioperatively. Bolier et al. opted to involve the neurotology team to remove the internal magnets of the CIs, proceed with surgery utilizing nonverbal communication with a computer monitor, and re-implantation of the magnets within 24 hours [10]. There still remains a paucity of data assessing the use of DBS in CI patients, and the variability with respect to the patient’s ability to effectively communicate without their CI may pose difficulties for the surgeon intraoperatively.

Intraoperative Neuromonitoring (IONM)

IONM consists of measuring several different types of evoked potentials during the surgery in order to localize and assess iatrogenic neuronal damage during the surgery. The electric potential difference, usually measured in voltage, induced by evoked potentials, can be destructive to brain implants and their surrounding tissue. Many manufacturers of CIs consider the use of transcranial electric stimulation to be contraindicated. However, there have been case reports of successful use of transcranial electrical stimulation in patients with CIs [11].

Studer et al. describe the case of a patient with Moebius syndrome manifesting as bilateral deafness status post CI and severe kyphoscoliosis requiring corrective spine surgery. They used transcranial electrical stimulation and measured evoked motor potentials with voltages that were contra-indicated by manufacturers but were shown to cause no harm to implants in cadaveric studies. The case report suggests that the use of IONM may be safe in the setting of CIs. Studer et al. discussed a 15-year-old patient with a left-sided CI who underwent corrective spinal surgery that included the use of transcranial electrical stimulation for motor-evoked potential monitoring [11]. In this case, the unchanged CI impedances following the case demonstrated a lack of tissue damage in the cochlea. The implants were also found to function normally 18 months post-surgery; however, more work will need to be performed to evaluate the safety of IONM use in patients with CI, and the neurophysiology team should always be made aware of the implant when placing cranial leads prior to the procedure.

Radiotherapy

Very little is currently known about the effects of radiosurgery on CIs. Kong et al. describe a 48-year-old woman with a left-sided CI and a recurrent meningioma [12]. She received a 26 Gy dose of radiation in a single fraction. Following the radiosurgery, the impedances of the implant were unchanged and auditory performance tests were stable. Similarly, other researchers have seen no issues with the use of up to 60 Gy radiation, although the consensus in the literature is that there is a low risk of implant failure as long as radiation doses are restricted to 40 Gy or less.

Cadaveric studies have been done to test for functional changes in CIs following irradiation at different doses. Although single doses over 20 Gy showed some initial functional changes of the cochlear implant, the function returned to normal just hours following the radiation dose. A high-dose (42.5 Gy) single-fraction linear accelerator-based radiosurgery demonstrated no sustained changes in CI integrity tests. With doses greater than 50 Gy, a loss of radio frequency link range occurred and rendered the implant dysfunctional requiring re-implantation in another study [13].

Monopolar Cautery

Monopolar cautery may pose a hypothetical risk for dysfunction of CIs, although no study has proven this to date. Generally, bipolar cautery is safe and favored at a distance >1 cm from the CI. Most manufacturers of CIs consider the use of monopolar electrical cauterization at or above the level of the second thoracic vertebra to be contraindicated. The use of monopolar cauterization may destroy the electronic implants and/or cause auditory nerve damage. However, cadaveric research has demonstrated that monopolar cauterization can sometimes be used without damage to CIs [14]. Studer et al. successfully performed T2 to L4 fusion in a patient with a CI and reported no adverse reaction to the patient’s hearing or the implant [11].

Intraventricular Shunt Placement

Programmable shunt valves are externally adjustable pressure differential shunts that can be adjusted through the external use of magnetic signaling. They therefore can be altered by other external magnetic fields which can cause dangerous changes to the amount of fluid being shunted through the system. Consequently, many CI manufacturers consider the presence of a programmable shunt valve to be a contraindication to cochlear implantation [15]. There are a few reports of cochlear implantation occurring in patients with programmable ventriculoperitoneal shunts. In these cases, the internal magnets of the CIs are placed away from the programmable shunt device. When placing CIs in patients with pre-existing shunts or when placing shunts in patients with CIs, it is important to make sure both the shunt tubing and implants are not injured, particularly when tunneling the shunt tubing or when preparing the initial skin flaps for cochlear implantation as they both occupy real estate on the calvarium, which may overlap [16]. To address this issue, we recommend contralateral insertion of ventriculoperitoneal shunt systems, or, if this is not feasible, at least 2 cm from the CI device. It is imperative that shunt settings are interrogated multiple times during the postoperative course, and if there is interference with the setting of the valve, removal and replacement may be required.

Discussion

Creating and delineating guidelines for neurosurgical interventions and diagnostic procedures in patients with CIs will ensure the safety of the patient and maintain the functionality of their life-changing implant, which provides them with the sense of hearing. The neurosurgeon must be mindful of the patient’s CI(s) and taking these points into consideration will ensure the patient is not subject to further procedures or hardship due to avoidable device failure. As technology continues to evolve, adverse effects on CIs due to routine neurosurgical procedures and diagnostic tests will likely decrease, and we recommend regular updates and case reporting to keep the community informed. Regarding MRIs, we recommend investigating whether a shorter, interval scan can be done in an awake patient using symptomatology as a guide on whether to proceed or not. This is complicated when patients require sedation or intubation. DBS, a procedure done in an awake patient, requires the device to be off for placement of the lead, complicating the communication and neurological examination intraoperatively. However, as more cases are reported, an efficient and effective workflow in the operating room may be established in order to optimize the patient’s feedback during the procedure. In addition, a preoperative plan may be established with the patient to better prepare them for this period of communication not aided by their CI. Next, the use of radiosurgery may need to be tailored to patients with CI in order to provide lower doses of radiation in a more fractionated course due to the potential complications of high-dose administration in a single fraction. Surgeons and radiation oncologists may be required to modify standard protocols to accommodate these unique patients. In regards to intraventricular shunt surgery, CIs may require the surgeon to favor contralateral shunt placement in a patient in which they would otherwise plan for ipsilateral placement. However, if an ipsilateral placement is required, additional preparation for burr hole placement and subcutaneous catheter tunneling may be required. During spinal surgery, the surgeon may not be required to forgo the important use of IONM, but it would be extremely helpful to encourage the publication of more data to demonstrate that the use of IONM is safe in these patients. Finally, in a similar manner, additional safety data will be critical to ensure the safe use of monopolar cautery in patients with CIs. Currently, while it is not clearly evident that monopolar cautery is harmful in these patients, few surgeons are currently willing to risk damage to the patient’s cochlear device.

In a recent shunt revision surgery at the authors’ institution, the issue of safe surgery in the setting of a CI arose, in which we only used bipolar cautery for hemostasis, which prompted this review and investigation. Additional data demonstrating safe surgery and procedures in CI patients will afford surgeons the assurance that they may proceed with their necessary surgical procedures as they normally would with monopolar cautery. As a greater number of patients with CIs undergo neurosurgical procedures, greater data will be obtained and the safety profile of CI in neurosurgery will assuredly be better defined. 

## Conclusions

With higher volumes of implantation occurring at younger ages, the chances that a CI patient will require neurosurgical care have increased. A review of the literature has identified a few conclusions. MRI compatibility remains debated, and evidence (although anecdotal) suggests that more modern models may be safer than older counterparts. If necessary, an MRI can be performed (preferably on a 1.5T scanner) and a tight headwrap should be placed; however, a small risk of dislocation of the magnet, and revision surgery may be present. DBS electrodes may be successfully placed after CI magnet removal and re-activated safely alongside a re-implanted CI. Discussing a communication strategy with the patient ahead of time will decrease anxiety and streamline the physical examination, assuring the accuracy and effectiveness of lead placement. Radiosurgery with <50 Gy is likely safe, but single fraction doses may need to be modified in CI patients. IONM and monopolar cautery have not been demonstrated to directly affect CIs; however, most devices still have them as contraindications, necessitating further testing of future devices. A programmable shunt valve should be placed on the contralateral side if possible, and an implantation plan may need to be modified to assure safe valve placement >2 cm away from CIs. Hopefully, these guidelines will assure the safety of these patients in the future and prompt additional investigation into the interactivity of these devices with common neurosurgical instruments and procedures. 
